# Fetus papyraceus causing dystocia in a rural setting: a case report

**DOI:** 10.1186/s13256-015-0666-9

**Published:** 2015-08-25

**Authors:** Dismas Matovelo, Edgar Ndaboine

**Affiliations:** 1 Department of Obstetrics and Gynecology, Catholic University of Health and Allied sciences, P.O. Box 1464, Mwanza, Tanzania; 2 Department of Obstetrics and Gynecology, Bugando Medical Centre, P.O. Box 1370, Mwanza, Tanzania

**Keywords:** Dystocia, Fetus papyraceus, Twin pregnancy

## Abstract

**Introduction:**

Fetus papyraceus is a rare condition which describes a mummified fetus in a multiple gestation pregnancy in which one fetus dies and becomes flattened between the membranes of the other fetus and uterine wall. We report a case of fetus papyraceus diagnosed during labor as a result of arrested descent.

**Case presentation:**

A 23-year-old Sukuma woman, gravida 2, para 1 presented to an maternity emergency unit of Sengerema Designated District hospital at a gestation age of 35^+5^ weeks as a referral from a rural health center due to arrested descent despite being in active labor for the past 8 hours. On vaginal examination, her cervix was 6cm dilated; fully effaced, presenting part was at station −3. A sharp and solid object-like thing was felt on the right side of her cervix. Due to uncertainty of the presenting part together with arrested descent, a decision was reached to deliver her by caesarean section. A twin gestation was identified during caesarean section: one being a male baby in cephalic presentation, weighing 1.9kg with Apgar score 8 in first minute and 9 in fifth minute with its own normal placenta and membranes. There was another atrophied placenta with calcifications without a cord and with mummified fetal bones on the anterior to the lower segment at the level of the internal orifice of the uterus. The atrophied placenta and mummified fetal bones weighed 200gms. One unit of blood was transfused intraoperatively due to severe anemia prior to surgery. Both the mother and the baby were discharged home in good condition.

**Conclusions:**

The primary concern for fetus papyraceus is its effect on the surviving fetus and on the mother. To avoid possible complications, the intrauterine diagnosis of fetus papyraceus by serial ultrasound examinations and routine placental examination to search for fetus papyraceus is mandatory.

## Introduction

The term fetus papyraceus is used when intrauterine fetal demise of a twin early in pregnancy occurs, with retention of the fetus for a minimum of 10 weeks resulting in mechanical compression of the small fetus such that it resembles parchment paper [1]. Fetus papyraceus is a rare complication with a reported incidence of one in 12,000 pregnancies [2] and between 1:184 and 1:200 twin pregnancies [3]. An antenatal diagnosis of a mummified fetus is usually an incidental finding during investigation of other pregnancy-related problems and it can be reached by ultrasonography [4]. Unfortunately in this case, no antenatal ultrasonography was done and referral was made due to prolonged labor and arrested descent of the fetus to a rural hospital in which ultrasound facilities were also unavailable.

The complications related to fetal papyraceus depend on whether it is a monochorionic or dichorionic twin pregnancy. Monochorionic twin pregnancies are associated with several complications when compared with dichorionic pregnancies [3, 4]. We present a case of a 23-year-old woman with a dichorionic twin pregnancy consisting of one normal fetus and one fetus papyraceus formation diagnosed during labor.

## Case presentation

A 23-year-old Sukuma woman, gravida 2, para 1, living 1 presented to the maternity emergency unit of Sengerema Designated District hospital at a gestation age of 35^+5^ weeks as a referral from a rural health center due to prolonged labor and arrested descent despite being in active labor for the past 8 hours. She was a housewife and had no formal education.

She had four unremarkable antenatal visits, in which she was normotensive in all her visits (blood pressure ranging from 120/75 to 110/60mmHg), she was negative for human immunodeficiency virus and syphilis and she had no history of any hereditary illnesses. Unfortunately she was not dewormed, not given intermittent presumptive treatment for malaria (IPT-sp), her hemoglobin level and hepatitis B status were not checked and no obstetrical ultrasound was performed during any of her visits.

**Fig. 1:**
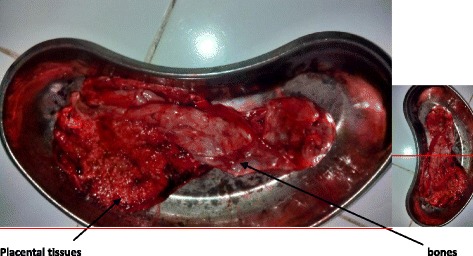
The atrophied placenta tissues and mummified fetal bones

Upon admission to the labor ward, her vital signs were: blood pressure of 120/60mmHg, pulse rate 76 beats/minute and respiratory rate of 20 breaths/minute. An obstetrical examination revealed a gravid abdomen with fundal height corresponding to 36 weeks gestation and four strong uterine contractions in 10 minutes, each lasting 40 seconds. The fetal heart rate was auscultated to be 142 beats/minutes by fetoscope. On digital vaginal examination, the cervix was 6cm dilated; fully effaced, presenting part was at station −3. A sharp and solid object-like thing was felt on the right side of her cervix. The object was felt to be solid and sharp/pointed with some areas of softness. Due to lack of ultrasound and uncertainty of the presenting part together with arrested descent a decision was reached to deliver her by caesarean section. She was informed about the findings which she accepted and she consented to caesarean delivery. A caesarean delivery under spinal anesthesia revealed a twin gestation: one being a male baby in cephalic presentation, weighing 1.9kg with Apgar score 8 in first minute and 9 in fifth minute with its own normal placenta and membranes. There was another atrophied placenta with calcifications without a cord and with mummified fetal bones anterior to the lower segment at the level of the internal orifice of the uterus. The atrophied placenta and mummified fetal bones weighed 200gms. Our conclusion of diamniotic dichorionic twin with fetal papyraceus in the layers of placenta was reached (Fig. 1).

She tolerated the surgery well, her estimated blood loss was 1000ml for which she received one unit of blood as her hemoglobin before caesarean was 7.2gm/dl. Her postoperative recovery was uneventful. On the seventh day postsurgery she was discharged home to be seen at the postnatal care clinic every week but unfortunately she was lost to follow up thereafter.

## Discussion

Fetus papyraceus is a rare complication resulting from failure to completely reabsorb the dead fetus in the second or third trimesters. The fetus will be compressed by its growing twin to a flattened, parchment-like state. The incidence of fetus papyraceus has been reported to be one in 12,000 pregnancies, [2] and ranges between 1:184 and 1:200 twin pregnancies [3]. The cause is usually unknown, but is associated with twin-to-twin transfusion, fetal genetic or chromosomal abnormalities and improper cord implantation such as velamentous cord insertion [3, 5].

The mummified fetus can be diagnosed early during antenatal care visits by imaging studies such as ultrasonography [4, 6, 7]. Unfortunately, our patient never had an ultrasound in all her prenatal visits which caused an intrapartum diagnosis of the condition. However, ultrasound can be faced with technical difficulties and affected by the anatomical position of the dead fetus. In the first trimester, this may present as vaginal bleeding especially if the fetus is completely absorbed and in most cases there are no complications afterwards. But in the second or third trimesters, it is usually associated with several complications such as preterm labor and hemorrhage as occurred in this patient, sepsis as a result of a dead fetus, consumptive hemorrhage and labor dystocia. These complications become severe when it is a monochorionic placenta rather than a dichorionic placenta [3, 4]. In this case, it was a dichorionic twin, which is why the complications encountered were prematurity, postpartum hemorrhage (PPH) and low birth weight.

In monochorionic twinning, the risk for cerebral palsy in surviving twin, aplasia cutis, and congenital malformations such as microcephaly or hydrocephalus are high [8–10]. A possible mechanism is the transfusion of thromboplastic proteins from the vanishing twin to the surviving twin, leading to disseminated intravascular coagulation (DIC). Researchers hypothesize that DIC results from reverse blood flow from the macerated twin to the viable twin, thus carrying thromboplastins into the circulation. This large thromboplastin load is hypothesized to lead to a state of DIC in the viable twin, which then leads to intrauterine central nervous system damage [7]. Another proposed mechanism for central nervous system damage involves large amounts of blood loss from the surviving twin to the low resistance system of the vanishing twin through placental anastomoses. This transfusion could cause wide fluctuation in intravascular pressures, leading to intraventricular hemorrhage which may results in cerebral palsy [9].

In this patient, the diagnosis of a mummified fetus was discovered during caesarean delivery following labor dystocia due to arrested descent and the detection of a sharp solid object-like thing during digital vaginal examination. The arrested descent of the fetus in this patient occurred as a result of low-lying fetus papyraceus which may have prevented the normal fetus to be delivered vaginally. Although other reports have shown a normal vaginal delivery is possible in cases like this [11], we opted for caesarean due to uncertainties encountered on vaginal examination. Another reason was the lack of access to ultrasound at our rural district hospital which forced us to conduct an emergency caesarean delivery [12]. Otherwise, with a low-lying fetal papyraceus without any fetal compromise to the surviving twin, the majority of patients will deliver vaginally after spontaneous onset of labor [11]. When fetus papyraceus is diagnosed early, expectant management with close fetal and maternal surveillance is advised [3, 4]. This could be done by performing serial ultrasound preferably every 2 to 3 weeks and monitoring of coagulation profile every fortnight.

## Conclusions

The primary concern of fetus papyraceus is its effect on the surviving fetus and on the mother. To avoid possible complications, the intrauterine diagnosis of fetus papyraceus by serial ultrasound examinations and routine placental examination to search for fetus papyraceus is mandatory.

## Consent

Written informed consent was obtained from the patient for publication of this case report and accompanying images. A copy of the written consent is available for review by the Editor-in-Chief of this journal.

## Abbreviation

DIC: Disseminated intravascular coagulation
